# The Hopfield-like neural network with governed ground state

**DOI:** 10.1186/1471-2202-14-S1-P257

**Published:** 2013-07-08

**Authors:** Leonid B Litinskii, Magomed Yu Malsagov

**Affiliations:** 1 Scientific Research Institute for System Analysis Russian Academy of Sciences, Moscow 119333, Russia

## 

Using a vector $u=\left(u_{1},...,u_{p}\right)$ let us construct a matrix $M_{ij}=\left(1-\delta_{ij}\right)u_{i}u_{j}$, i,j=1,..,p, where $\delta_{ij}$ is the Kronecker delta, $u_{i}\in R^{1}$ and ${∥u∥}^{2}=p$. We define a Hopfield-like neural network with a connection matrix $J_{ij}=\left(1-2x\right)M_{ij}$ proportional to $M=\left(M_{ij}\right)$ and threshold $T_{i}=q\left(1-x\right)u_{i}$ proportional to coordinates $u_{i}$. Real quantities  x and q are our free parameters. The dynamics of the network is defined by the equation $s_{i}\left(\tau +1\right)=\text{sgn}\;\left(\sum_{j=1}^{p}J_{ij}s_{i}\left(\tau \right)+T_{i}\right)$, where $s_{i}\left(\tau \right)=\pm 1$ are binary coordinates of the configuration vector $s\left(\tau \right)=\left(s_{1}\left(\tau \right),...,s_{p}\left(\tau \right)\right)$ describing the state of the network at the given time  τ. Fixed points of the network are local minima of the energy. The configurations providing for the global minimum are called the ground state. Just the ground state is usually associated with the memory of the network. It turns out that to a considerable extent the ground state of our network can be governed by the parameters  x, q and  u. The point is that in full the energy E(s) is defined by the scalar product of vectors  s and  u: $E\left(s\right)\sim -\left(1-2x\right){\left(u,s\right)}^{2}-2\left(1-x\right)q\left(u,s\right)$. Then the number of different values of the energy is equal to the number of different values of the cosine $\text{cos}w=\left(s,u\right)/p$ when  s ranges over all $2^{p}$configurations. Let us arrange different values of cosines in the decreasing order starting the numeration from 0: $\text{cos}w_{0}$ >$\text{cos}w_{1}$ > ...>$\text{cos}w_{t}$. The set of all the configurations for which the cosine is equal to $\text{cos}w_{k}$ we define as the class ${\text{Σ}}_{k}$: ${\text{Σ}}_{k}=\left\{s:\;\left(s,u\right)=p\cdot \text{cos}w_{k}\right\}$. It is easy to see that for each  k the equalities ${\text{Σ}}_{t-k}=-{\text{Σ}}_{k}$ and $\text{cos}w_{t-k}=-\text{cos}w_{k}$ are fulfilled. The following statement is true:

**Theorem**. As  x increases beginning from the initial value equals to 0, the ground state of the network coincides in consecutive order with the classes ${\text{Σ}}_{k}$: ${\text{Σ}}_{0}\to {\text{Σ}}_{1}\to {\text{Σ}}_{2}\to ...\to {\text{Σ}}_{k_{\text{max}}}$. The transition from ${\text{Σ}}_{k-1}\to {\text{Σ}}_{k}$ takes place in the critical point $x_{k}=\frac{q/p+\left(\text{cos}w_{k-1}+\text{cos}w_{k}\right)/2}{q/p+\text{cos}w_{k-1}+\text{cos}w_{k}},\;k=1,2,..k_{\text{max}}$, and while $x\in \left(x_{k},x_{k+1}\right)$ the ground state of the network is the class ${\text{Σ}}_{k}$. The transitions ${\text{Σ}}_{k-1}\to {\text{Σ}}_{k}$ ceases when the denominator of the expression for $x_{k}$ becomes negative. If q/p>2, then $k_{\text{max}}=t$.

In large part this theorem allows one to regulate the ground state of the network. Let us examine  p-dimensional hypercube whose side length is 2 and the center is in the origin of coordinates. The configurations  scoincide with vertices of the hypercube. Possible symmetric directions of the hypercube have to be chosen as vectors  u. For each choice of  uthe configurations  sare distributed symmetrically around this vector. Each such symmetrical set of configurations is one of the classes ${\text{Σ}}_{k}$, and using the theorem one can do it the ground state of the network. In particular, we can construct the ground state with very large number (~$C_{p}^{k}$) of configurations. If nonzero components of the vector  uequal in moduli, for each  x the only fixed points of the network are its ground state. The classification of all possible applications of this Theorem is not yet finished.

Computer simulations show that basins of attraction of such fixed points are very small. It is not surprising, since the number of the fixed points is very large, and the volume of each basin of attraction is of the order of the volume of the unit hypersphere divided by the number of fixed points.

